# The implication of the shortage of health workforce specialist on universal health coverage in Kenya

**DOI:** 10.1186/s12960-017-0253-9

**Published:** 2017-12-01

**Authors:** Mumbo Hazel Miseda, Samuel Odhiambo Were, Cirindi Anne Murianki, Milo Peter Mutuku, Stephen N. Mutwiwa

**Affiliations:** 1 MOH/JICA OCCADEP Project, Nairobi, Kenya; 2 The Palladium Group, Afya Pwani Project, Nairobi, Kenya; 3 Human Resource Development Unit, Ministry of Health, Nairobi, Kenya; 4 USAID FUNZOKenya Project, IntraHealth International Inc., Nairobi, Kenya; 5 Jhpiego, Nairobi, Kenya

**Keywords:** Health workforce, Shortage, Specialists, Training

## Abstract

**Background:**

Globally, there is an acute shortage of human resources for health (HRH), and the greatest burden is borne by low-income countries especially in sub-Saharan Africa and some parts of Asia. This shortage has not only considerably constrained the achievement of health-related development goals but also impeded accelerated progress towards universal health coverage (UHC). Like any other low-income country, Kenya is experiencing health workforce shortage particularly in specialized healthcare workers to cater for the rapidly growing need for specialized health care (MOH Training Needs Assessment report (2015)). Efficient use of the existing health workforce including task shifting is under consideration as a short-term stop gap measure while deliberate efforts are being put on retention policies and increased production of HRH.

**Methods:**

The Ministry of Health (MOH) with support from the United States Agency for International Development-funded FUNZOKenya project and MOH/Japan International Cooperation Agency (JICA) project conducted a country-wide training needs assessment (TNA) to identify skill gaps in the provision of specialized health care in private and public hospitals in 46 out of Kenya’s 47 counties between April and June 2015. A total of 99 respondents participated in the TNA. Structured questionnaires were used to undertake this assessment. The assessment sought to determine the extent of skill gaps on the basis of the national guidelines and as perceived by the County Directors of Health (CDH). The questionnaires were posted to and received by all the respondents a week prior to a face-to-face interview with the respondents for familiarization. Data analysis was done using SPSS statistical package.

**Results:**

Overall, the findings revealed average skill gaps on selected specialists (healthcare professional whose practice is limited to a particular area, such as a branch of medicine, surgery, or nursing, especially, one who by virtue of advanced training is certified by a specialty board as being qualified to so limit his or her practice, Free dictionary) at 85 and 62% when compared to the guideline and as perceived by the CDH respectively. It also revealed that gynecologists exceeded the requirements by 88 and 246% against the guidelines and as perceived by the CDH respectively.

**Conclusion:**

There is an overall huge gap in health specialists across the 46 counties, and the focus of training should be on the following specialists: cardio-surgeons, neurosurgeons, oncologists, nephrologists, lung and skin clinical officers, anesthetic clinical officers, cardiology nurses, forensic nurses, dental nurses, accident and emergency nurses, and oncology nurses. More innovative approaches, including the use of technology, need to be considered to address this challenge in the immediate, medium, and long terms. Policies and legal frameworks should be developed to facilitate cross-county sharing of specialist expertise. Efforts need to be made to ensure harmonized skill gaps revealed by the guideline and as perceived by the CDHs to inform the development of mitigation strategies.

## Background

Globally, the shortage of healthcare workers is projected to reach 12.9 million by 2035. Currently, that figure stands at 7.2 million. The WHO report (2013) at the Third Global Forum on Human Resource for Health indicated that, if not addressed, the shortages identified in the report will have serious implications on the health of billions of people across the world [1]. Although nearly half of the world’s population resides in rural areas, only 38% of the world’s nurses and 25% of the doctors work in these areas [2].

The shortage of healthcare workers is more prevalent in developing countries. The dire shortage of healthcare workers has considerably constrained achievement of health-related Millennium Development Goals (MDGs) [3] and if not addressed may undermine the achievement of health-related Sustainable Development Goals (SDGs).

This shortage of healthcare workers in many countries, including developed countries such as the United Kingdom and United States of America, is attributable to a number of factors such as an increase in chronic diseases and conflict, brain drain in the case of developing countries, and concentration of such workers in urban areas. Resolving the problem of inadequacy of the health workforce will require a united effort by national and international agencies [3].

About 57 countries, the majority being in Africa and Asia, face severe healthcare workers crisis. The World Health Organization (WHO) estimates that at least 2 360 000 health providers and 1 890 000 management support workers are required to fill the existing gap.

Healthcare workers are inequitably distributed with severe imbalances between developed and developing countries, with the latter most adversely affected. Sub-Saharan Africa, with 11% of the world’s population, 24% of the global disease burden, and only 3% of the world’s health workers [4], faces the greatest challenge.

In Kenya, the total number of the health workers currently employed in the County Departments of Health as well as in the public, faith-based organization (FBO), and private-for-profit health facilities is estimated at 31 412 (Training Needs Assessment, 2016)^3^. These numbers are below the required of 138 266 healthcare workers as per the Norms and Standards Guidelines by the Ministry of Health.

In order to offer better health services in Kenya, the Health Sector Strategic Plan III (2012–2017) structured service delivery into four main tiers: tier 1, community; tier 2, primary care level; tier 3, county level; and tier 4, national level. The Kenya Health Policy defines eight policy imperatives^2^ including health infrastructure. To guide the development of a robust health infrastructure, the Ministry of Health reviewed and developed Norms and Standards for infrastructure of human resource for health based on the defined population coverage by tiers. Tier 1 covers a population of 5000 people, a dispensary covers 10 000 people, a health center covers a population of 30 000 people, a primary hospital covers 100 000 people, and a secondary care hospital covers a population of one million, while a national teaching and referral hospital covers a population of five million people. It also took into consideration the complexity and the degree of specialized care reflected at each level of the defined tier.

In the wake of devolution which is a form of decentralization that created two levels of government with distinct and complimentary functions^1^ at national and sub-national, health service delivery and to some extent specialized health care became functions of the county government prompting the need to establish the capacity of the counties’ human resource requirement to effectively and efficiently deliver these services.

Benchmarking the human resource for health (HRH) capacity with the WHO requirement and against the capacity of the health training institutions in Kenya is considered essential as it enables the government to forecast its workforce, the past inflow, and expected inflow, thus assisting in effective planning and distribution as dictated by needs.

The Ministry of Health and development partners of health in Kenya undertook training needs assessment (TNA) in September 2015, which assessed the overall gap of health specialists as per the standard numbers that have been set as well as the ideal numbers as perceived by the County Directors of Health (Table 1). This assessment sought to identify the health specialists in 94 county referral hospitals. The service domain included surgery, radiology, central sterile services department (CSSD), medical laboratory, ICU, and renal dialysis.

**Table 1:** A summary of the training needs assessment report tables

The training needs assessment report table showing the gap of health workforce specialists in Kenya									
46 counties analyzed	Total standard number	Total employment	Percent shortage by standard	Total ideal number	Percent shortage by ideal	Mean of employed staff per county	Ideal mean per county	*t* test *P* value [comparing employed with ideal]	*t* test *P* value [compare employed with standards]
CDH	138 266	26 763	81	67 312	60	683	1599	< 0.000 1	< 0.000 1
MOH	6 428	1 719	73	3 942	56	16	21	0.029 1	< 0.000 1
Anesthetist	590	106	82	386	73	2	9	< 0.000 1	< 0.000 1
ENT surgeons	285	30	89	205	85	1	4	< 0.000 1	< 0.000 1
Ortho surgeons	285	29	90	188	85	1	4	< 0.000 1	< 0.000 1
Cardio surgeons	10	0	100	120	100	0	3	< 0.000 1	0.000 7
Neuro surgeon	10	4	60	1 140	100	1	2	< 0.000 1	0.135
Urolo surgeons	10	3	70	115	97	0	3	< 0.000 1	0.036 5
Pediatrics surgeons	10	8	20	139	94	0	3	< 0.000 1	0.646 1
Neurologists	275	4	99	114	96	0	2	< 0.000 1	0.135
Plastic surgeons	10	14	− 40	141	90	0	3	0.000 5	0.708 9
Obstetricians/gynecologists	560	1 054	− 88	305	− 246	2	7	< 0.000 1	< 0.001
General internists/physicians	570	1 719	− 202	3 982	57	36	86	0.079 86	< 0.001
Cardiologists	20	1	95	110	99	0	2	< 0.000 1	< 0.001
Chest specialists	0	1	5813	5 813	100	0	3	< 0.000 1	0.151 5
Endocrinologists	0	1	95	95	99	0	2	< 0.000 1	0.32
Gastro-enterologists	20	4	80	105	96	0	2	< 0.000 1	0.013
Graduate clinical officers	4 585	939	80	1 807	48	20	40	0.084 9	< 0.001
CO ENT	1 375	109	92	490	78	2	11	< 0.000 1	< 0.001
CO lungs and skin	1 885	208	89	532	61	5	12	< 0.000 1	< 0.001
CO ophthalmology/cataract surgery	1 080	90	92	338	73	2	8	< 0.000 1	< 0.001
Co pediatrics	1 395	135	90	552	76	3	12	< 0.000 1	< 0.001
Anesthetist nurses	1 630	61	96	696	91	1	15	< 0.000 1	< 0.001
Accident and emergency care nurses	2 750	13	100	482	97	0	10	< 0.000 1	< 0.001
BSN nurses	1 180	1 189	−1	2 426	51	26	54	0.022 5	0.990 5
KRCHN	41 732	8 612	79	21 635	60	17	494	< 0.000 1	< 0.001
Community public health nurses (KECHN)	77 544	796	99	1 611	51	47	66	0.608 4	< 0.001
Cardiology nurses	20	0	100	483	100	0	10	< 0.000 1	0.000 6
Total nationally	282 525	43 612	85	115 264	62				

## Objective of the TNA

The objectives of the TNA are to determine the overall gap of specialized healthcare provision at all levels of the health system and to provide recommendations to address these gaps.

## Methods

The Constitution of Kenya (2010) established two levels of government: the national government and the 47 devolved county governments. Under this new arrangement, the national government has been assigned the function of policy, capacity building, and national referral hospitals while the 47 county governments focus on county health delivery. The constitution further introduced health care as a fundamental basic right every citizen must have access to the highest attainable standards. To facilitate realization of this right, the national governments undertook a TNA to establish the capacity of the county governments to deliver on their new mandate. The assessment sought to determine the extent of the overall gaps of specialized care delivery as compared to the defined optimal requirement in the national guideline and as perceived by the County Directors of Health who are in charge of service delivery in the counties.

This TNA was conducted under the leadership of the Director of Human Resources Development based at the Ministry of Health at the national level. A Steering Committee with clear terms of reference was constituted by the Director of Medical Services (DMS) to oversee the assessment process.

This study covered all specialist staff at post-basic, which refers to health workers undertaking specialist training after their diploma or bachelor’s degree level currently posted in county (Government of Kenya, FBO, and private-for-profit) hospitals, the numbers required for effective service delivery, and the gaps (by facility, facility levels L4 and L5, and county). This TNA considered functions and job responsibilities, rather than occupations, as the basis for determining required competences and training needs. Kenya Essential Package for Health (KEPH) defines, for each intervention, the “lowest cadre” expected to carry out the intervention and the lowest facility level. It is however on which specialized professional categories should provide them.

A total of 99 respondents participated in the TNA. These comprised medical superintendents of level 4 and 5 county referral hospitals, health directors of FBO, and chief executive officers (CEO) of private hospitals. The other respondents were directors from the national referral facilities of Moi and Kenyatta Referral Hospitals as well as the medical superintendent of Mathari National Mental Referral Hospital. These respondents were included in the assessment because of the leadership role they play in these key institutions. The others interviewed included the County Directors of Health of 46 counties. The assessment used structured questionnaires that were sent to respondents for familiarization followed by a face-to-face interview by the enumerators.

Primary data was collected by administering seven structured questionnaires to the respondents. Desktop review was conducted for secondary data. The data collection took a month. The Ministry grouped the country into 11 clusters each consisting of 3 to 10 counties depending on the geographical distribution, namely, Lake Basin, Western, North Rift, South Rift, Central, Eastern, North Eastern, Coast, Central, and Eastern regions, which were divided into two clusters each because of the vast landscape within the counties. Each cluster had a supervisor in charge of data collection while each County Department of Health provided two enumerators who collected the data. Collection of data in each county took 2 to 3 days depending on the availability of the respondents. During the assessment, the County Directors of Health were asked to provide the actual number of staff employed by their departments as well as their perceived ideal number of the staffs required based on the workloads.

Enumerators and supervisors were recruited and trained on data collection. The training included familiarization with the questionnaires and instructions on administration of the tool based on the protocol and their job descriptions. A pre-test of the questionnaire was conducted as part of the training. Based on the feedback from the pre-test, the questionnaires were revised and finalized.

## Limitation of the study

The ideal number of health workforce suggested by the County Directors of Health was based on perception and could have had some bias.

## Analysis

Data entry and analysis was done using SPSS statistical package. As a first step, open questions had to be placed into categorical variables. The data entry and analysis process was guided by established standards of monitoring and evaluation. The United States Agency for International Development (USAID)/FUNZOKenya and Japan International Cooperation Agency (JICA) provided technical leadership in data analysis and reporting.

Descriptive analysis was used in computing frequencies, recoding variables, and running cross tabulation. Frequency counts and percentages were the main statistical tools employed in analyzing the data generated through the questionnaires. A non-response to any item of the questionnaires was considered invalid and excluded from the analysis. Data outputs were organized by question and by the variables described in the expected outputs in section 5 (e.g. county, cadre, level of care, subsector).

During the process of report writing, further analysis was carried out as needed. Tables, graphs, and pie charts were created using the analysis outputs. The graphs were used to illustrate frequencies and therefore occurrences of events or activities.

The draft TNA report was shared with the stakeholders at a meeting held between 19 and 20 November 2015 and feedback included in the final report. The stakeholders comprised of MOH-HRD, MOH technical departments, counties, training institutions, and development partners.

## Results

Human Resources for Health Norms and Standards Guidelines stipulate the number of healthcare workers required by cadre at different tiers of the health sector for effective health service delivery in Kenya. This Guideline further provides the basis for rational and equitable distribution of the health workforce across the different tiers/levels of healthcare delivery in the country.

The TNA data were aggregated and analyzed by cadres at national and county levels and compared with the Norms and Standards Guidelines. The specialized staff distribution and availability was further analyzed according to their functions in either direct service delivery or administration.

In general, the findings reveal that on average, specialists’ skill gaps range between 85 and 62% when compared to the Norms and Standards and as perceived by the CDH respectively. However, on average, gynecologists, plastic surgeons, and Bachelor of Science in Nursing (BSN) have surpassed the required level by more than 100% when compared with the national guideline. Specifically, gynecologist have exceeded the requirements by 88 and 246% against the guidelines and as perceived by the CDH respectively. This finding should inform further investment in training of gynecologist/obstetrician in the medium term. Further in-depth study should be undertaken to establish the reason for the high number of gynecologists beyond the recommended levels.

The study also revealed that physicians are 57% less than the “ideal” number suggested by the County Directors of Health but thrice as much the recommended number by Norms and Standards Guidelines. There is need for further studies to shed more light on the reasons for the variance in shortage across the entire cadre spectrum as revealed by the perception of the County Directors of Health and the national guidelines.

### Gaps in specialists

#### Medical officer specialists

The skill gaps among medical officer specialists is significant when compared to the national guideline and the reported ideal numbers suggested by the County Directors of Health (CDH). Findings of sampled specialty areas are detailed as follows: neurosurgeons had a gap of 100 and 60% against the guidelines and as suggested by the CDH respectively; there was no significant difference between the gap as reflected by the MOH Norms and Standards Guidelines as compared to the perceived number by the CDH (95% and 99%). The shortage of urologist accounted for 70 and 97% against the Norms and Standards Guidelines and the ideal numbers by the CDH, respectively, while neurologists had a shortage of 99% against the Norms and Standards Guidelines and 96% against the ideal numbers by the CDH. Pediatric surgeons had a shortage of 20% against the Norms and Standards Guidelines and 94% against the ideal number by the CDH respectively.

Overall, there is a big variance in the gaps between the Norms and Standards Guidelines and the ideal numbers by the CDH that need to be interrogated. The cardiologists and endocrinologists both had a shortfall of 99% of the health workforce against ideal number suggested by the CDH while the cardiologists had a shortfall of 95% against the Norms and Standards Guidelines. Endocrinologists like chest specialists are not reflected in the Norms and Standards Guidelines. Gastro-enterologists had a shortfall of 96% against the ideal number of workforce suggested by the CDH while the shortage against the Norms and Standards Guidelines is 80%. The ortho surgeons had a shortfall of 90% healthcare workers against the Norms and Standards Guidelines and 85% against the ideal number suggested by the CDH. Ear, nose, and throat surgeons had a shortage of 85% against the ideal number suggested by the CDH and 89% against the Norms and Standards Guidelines. And lastly, anesthetists had 82% shortage of the health workforce against the Norms and Standards Guidelines and 73% against the ideal numbers suggested by the CDH. It is interesting to note that there is inconsistency in the required number of plastic surgeons against the Norms and Standards Guidelines in which there are 14 times more plastic surgeons against the ideal number suggested by the CDH while against the Norms and Standards Guidelines, there is a surplus of four specialists.

The gaps among other healthcare workers who are working closely with medical officers are detailed below.

#### Clinical officer (medical assistant) specialists

A sample of specialized skills was analyzed for this cadre. The findings revealed specialist skill gaps among the clinical officers in the country as follows: overall, the gap for clinical officers who had undertaken a post graduate specialized training was 80% against the national guidelines compared to 48% against the ideal numbers suggested by the CDH. Specifically, the gap for ear, nose, and throat (ENT) was 92% against the national guideline compared to 78% against the ideal numbers suggested by the CDH while in lungs and skin speciality, the gaps were 89 and 61% against the guidelines and as perceived by the CDH respectively. Regarding ophthalmology/cataract surgery, the gap against the guideline was 92 and 73% as perceived by the CDH. Regarding specialization in pediatrics, gaps at 90% according to the Norms and Standards Guidelines and at 76% as perceived by the CDH were noted.

#### Nurse specialists

Skill gaps among the nurses for sampled specialized health service delivery areas were also noted in the following areas: anesthetist nurses at 96% against the Norms and Standards Guidelines while at 91% as suggested by the CDH; trauma and emergency care nurses at 100% against the Norms and Standards Guidelines and at 97% as suggested by the CDH; on the contrary, BSN has exceeded the Norms and Standards Guidelines by 1% but in short supply by 60% as perceived by the CDH; KRCHN has a shortage of 79% against the guideline while it has a deficit of 60% according to the CDH; cardiology nurses had a shortage of 99 and 100% respectively against the Norms and Standards Guidelines and the ideal numbers by the CDH respectively. There were no nurse oncologists in the counties.

## Discussions

Kenya’s specialized healthcare skill gaps pose a major threat to the delivery of specialized health care. The policies and strategies to address these shortcomings need to be an integral part of the health systems’ strengthening agenda. This is now more critical if Kenya is to make significant progress towards attainment of universal health coverage, health-related Sustainable Development Goals, and realization of right to health care as envisioned in the Constitution 2010. Ironically, Kenya recently launched a mega $420 million state-of-the-art medical equipment project across the 47 counties in all the public hospitals with the aim of improving access to specialized healthcare services. The current incongruence on the specialized health workforce and the ongoing upgrading of equipment and infrastructure is a manifestation of weak health system approach to the sector development.

In December 2012, the United Nations General Assembly adopted Resolution A/RES/63/33, giving further political impetus to universal health coverage and recognizing the need for an “adequate, skilled, well trained and motivated workforce.” However, the World Health Report (2006)—“Working together for Health” and several other reports since then have highlighted unacceptable variations in availability, distribution, capacity, and performance of human resources for health and identified countries below a critical threshold of health workers, deemed to be generally necessary to achieve high coverage of essential health services.

The evidence presented here show that Kenya lags behind on the supply of the needed specialist healthcare workers to help her in both the short and medium terms. The UNICEF report “Addressing the Health Worker Shortage: A critical action for improving maternal and new born Health,” shortages of skilled healthcare workers arise from many factors. These include underinvestment in training and recruitment, weak incentive structures, systems and mechanisms for healthcare workers, and migration of skilled healthcare workers from developing countries to industrialized nations triggered by the high health worker demand in industrialized countries.

With the coming of devolution, HRH management function was rapidly transferred to counties before appropriate county-level structures and adequate capacity to undertake these functions were in place. There was also lack of clarity over specific roles and responsibilities at county and national government and of key players at each level. Subsequently, health worker strikes and mass resignations were witnessed (Tsofa et al. [5]). The factors triggering this exodus include low pay, poor working conditions, and perceived “hostility” from the new employer, the county government. This disquiet has been felt across the cadres resulting in protracted industrial protests by the nurses late 2016 and part of mid-2017 agitating for better working conditions. While the government was swift to address the nurses’ concerns, it was so short lived that they are back to the streets for several months. The doctors’ strike extended and almost crippled healthcare services for more than 100 days before finally agreeing on a return-to-work formula.

While Kenya trains majority of its health workforce locally, especially at undergraduate level, a good number obtain their post graduate training overseas. As a requirement, upon returning to the country, they have to undergo local clearance process before registration by their respective regulatory bodies. Records show that The Kenya Medical Practitioners and Dentists Board (KMPDB) had registered around 9000 medical doctors and 1000 dentists over the past 32 years, but only 75% of these are currently considered “active,” having renewed their medical licenses within the past 5 years, and 11% of the active medical doctors are 61 years of age or older, while an additional 17% are 51–60 years old, although many will continue working after retirement age.

In an effort to enhance efficiency in HRH management especially healthcare specialists between the national and county governments, the two levels of governments have established institutions and mechanisms to facilitate sharing of the scarce specialized skills. These mechanisms include intergovernmental consultative forums and intercounty HRH forums. The Third Global Forum on Human Resources for Health provided an opportunity for an inclusive dialog with many stakeholders involved in efforts to develop and effectively manage human resources globally and specifically in low-income countries.

Key highlights from the training needs assessment reveal that a total of 31 412 healthcare workers are employed in the County Departments of Health, FBOs, and private-for-profit health facilities while the Norms and Standards Guidelines recommend 138 266 and the ideal number expected as suggested by the CDH is 67 312. This is with exception of Baringo County which was however excluded due to logistical concerns. This shows that there is a shortage of 81% health workforce against the Norms and Standards and 60% against the ideal numbers suggested by the CDH at the county level. This means that delivery of Kenya’s Essential Package for Health (KEPH) is jeopardized due to inadequate staffing levels especially in counties such as Turkana, Uasin Gishu, Wajir, Kajiado, and Garissa which operate at staffing levels below 10 (Ministry of Health TNA Report [6]). There are no cardiologists, oncologists, and nephrologists employed in the counties. Clinical officers are least employed in Samburu, Nairobi, Wajir, Bomet, and Lamu counties. Nurses specialized in forensics, dentistry, and accident and emergency are the fewest in the counties while there is no nurse specialized in oncology.

## Conclusion

What emerged from this assessment is that Kenya is experiencing a severe shortage of health specialist which is likely to jeopardize her efforts towards realization of rights to health and a threat to the attainment of Sustainable Development Goals (SDGs). Therefore, there is an urgent need for policies and strategies that provide a holistic approach to human resources development and management in the short, medium, and the long terms to bridge the existing huge skill gaps especially for the specialists in the 47 counties. The already established mechanisms like the intergovernmental consultative forums, for addressing HRH challenges in the counties, need to be strengthened and scaled up. To be considered in these policies and strategies is the specific roles played by each cadre and the use of technology in the national and health sector development as Kenya strides to join the league of upper middle-income countries. The question of whether development of specialists in Kenya should be left to market forces or whether some level of government intervention needs to be introduced is a debate that needs to be ignited as soon as possible. This debate is at the backdrop of significant investment and over supply of some specialized areas (gynecologists/obstetricians).

More specifically, the focus on the training of specialists should be cardio-surgeons, neurosurgeons, oncologists, nephrologists, lung and skin clinical officers, anesthetic clinical officers, and cardiology nurses, forensic nurses, dental nurses, accident and emergency nurses, and oncology nurses. On the other hand, from the findings, the least number of health specialists required for effective service delivery are pathologists, epidemiologists, and dermatologist. This should be noted by the Human Resource Development Unit of the Ministry of Health when doing training projections.

Given the disparity in the Norms and Standards Guidelines and perception of the County Directors of Health in the ideal number of specialists required of service delivery, there should be an all-inclusive and rational review of the norms and standards to include the views of the County Directors of Health.

## Abbreviations

CDH: County Directors of Health
CDoH: County Department of Health
DMS: Director of Medical Services
FBO: Faith-based organization
GoK: Government of Kenya
HRD: Human Resource Development
HRH: Human resource for health
KEPH: Kenya Essential Package for Health
KHSSP: Kenya Health Sector Strategic and Investment Plan
MoH: Ministry of Health
SPSS: Statistical Package for the Social Sciences
TNA: Training needs assessment
UHC: Universal health coverage

## Footnotes

3
http://www.health.go.ke, Email:ps@health.go.ke

2 Kenya Health Policy 2014-20130, Ministry of health, afya house, cathedral road, http://www.health.go.ke

1 Kenya Law report—Constitution of Kenya, 2010
